# The Impact of Stannous, Fluoride Ions and Its Combination on Enamel Pellicle Proteome and Dental Erosion Prevention

**DOI:** 10.1371/journal.pone.0128196

**Published:** 2015-06-01

**Authors:** A. A. Algarni, M. C. M. Mussi, E. B. Moffa, F. Lippert, D. T. Zero, W. L. Siqueira, A. T. Hara

**Affiliations:** 1 Oral Health Research Institute, Indiana University School of Dentistry, Indiana University, Indianapolis, Indiana, United States of America; 2 Department of Biochemistry, Schulich School of Medicine & Dentistry, The University of Western Ontario, London, Ontario, Canada; UW Medicine Neuropathology, UNITED STATES

## Abstract

**Objectives:**

To compare the effects of stannous (Sn) and fluoride (F) ions and their combination on acquired enamel pellicle (AEP) protein composition (proteome experiment), and protection against dental erosion (functional experiment).

**Methods:**

In the proteome experiment, bovine enamel specimens were incubated in whole saliva supernatant for 24h for AEP formation. They were randomly assigned to 4 groups (n=10), according to the rinse treatment: Sn (800ppm/6.7mM, SnCl_2_), F (225ppm/13mM, NaF), Sn and F combination (Sn+F) and deionized water (DIW, negative control). The specimens were immersed 3× in the test rinses for 2min, 2h apart. Pellicles were collected, digested, and analyzed for protein content using liquid chromatography electrospray ionization tandem mass spectrometry. In the functional experiment, bovine enamel specimens (n=10) were similarly treated for pellicle formation. Then, they were subjected to a five-day erosion cycling model, consisting of 5min erosive challenges (15.6 mM citric acid, pH 2.6, 6×/d) and 2min treatment with the rinses containing Sn, F or Sn+F (3×/d). Between the treatments, all specimens were incubated in whole saliva supernatant. Surface loss was determined by profilometry.

**Results:**

Our proteome approach on bovine enamel identified 72 proteins that were common to all groups. AEP of enamel treated with Sn+F demonstrated higher abundance for most of the identified proteins than the other groups. The functional experiment showed reduction of enamel surface loss for Sn+F (89%), Sn (67%) and F (42%) compared to DIW (all significantly different, p<0.05).

**Conclusion:**

This study highlighted that anti-erosion rinses (e.g. Sn+F) can modify quantitatively and qualitatively the AEP formed on bovine enamel. Moreover, our study demonstrated a combinatory effect that amplified the anti-erosive protection on tooth surface.

## Introduction

Fluoride and stannous ions have shown anti-erosive action *in vitro and in situ* [1]. Topical application of fluoridated rinses (112–450 ppm F, as NaF) may protect against erosive tooth wear (ETW) [2] by forming a CaF_2_ or CaF_2_-like layer on the enamel surface [1]. Similarly, stannous ion-containing rinses (800 ppm Sn, as SnF_2_ or SnCl_2_) seem to prevent ETW by depositing a stable acid-resistant layer on the tooth surfaces [3–5]. Sn-containing rinses may also react with dental hard tissues, due to the low pH, leading to the incorporation of the Sn ion into the enamel, thereby creating a more acid-resistant substrate [6].

The acquired enamel pellicle (AEP), a protein-based adsorbent layer, has been shown to reduce ETW by physically preventing the acid from contacting the tooth surfaces [7]. *In vitro* and *in situ* studies have reported that the composition, thickness and maturation stage of the AEP regulate its protection against ETW [7, 8], which is also affected by the erosive acid concentration and exposure time [9, 10]. Some studies have suggested that AEP may interact with anti-erosive agents, such as F and Sn, modifying their protective effects [11, 12]. Fluoride precipitates (CaF_2_) were found to be more stable and protective in the presence of AEP [13]. Similarly, increased protection has been reported for dental surfaces treated with Sn rinses, when associated with the AEP [14].

In this study, we hypothesized that AEP treated with F and/or Sn ions solutions can provide different proteome and lead to different levels of protection against erosion. In order to test these hypotheses, we: 1) identified and characterized the protein composition of the AEP formed in the presence of F, Sn and Sn+F ions by using mass spectrometry instrumentation; and 2) investigated the ability of the selected ions to prevent erosion on enamel by employing an *in vitro* demineralization model in presence of AEP formed from whole saliva supernatant.

## Materials and Methods

### Experimental design

This study protocol was reviewed and approved by the Indiana University Purdue University of Indianapolis IRB, #130210786. It was conducted in two independent experiments: proteome and functional. In the proteome experiment, bovine enamel slabs (n = 8) were exposed to whole saliva supernatant for the AEP formation followed by treatment with the specific tested rinses. AEP proteins were harvested from the enamel surface and subjected to mass spectrometry and bioinformatic analyses. The functional experiment tested the effects of rinse treatments: Sn+F, F, Sn and deionized water (control group). Bovine enamel slabs (n = 10) were submitted to a demineralization-cycling protocol. The response variable was surface loss (μm), measured at the end of the cycling procedure. Both experiments followed the complete randomized design.

### Solutions preparation

The Sn solution contained 6.7 mM Sn (SnCl_2_, CAS#7772-99-3, Sigma-Aldrich, St. Louis, MO, USA) stabilized by sodium gluconate (AC18139, FisherSci, Fair Lawn, NJ, USA). The NaF solution contained 13 mM F (NaF, CAS#7681-49-4, Sigma-Aldrich, St. Louis, MO, USA). Sn+F combined both solutions (6.7 mM Sn as SnCl_2_ and 13 mM F as NaF). All solutions were pH-adjusted to 4.5 using HCl or KOH. The erosive solution used was 15.6 mM citric acid (C1857, Sigma-Aldrich, St. Louis, MO, USA), pH 2.6.

### Saliva collection

Stimulated human whole saliva was collected from six healthy donors without active caries, periodontal disease or hyposalivation, and not taking any medications. In order to participate in the study, the donors had to sign an IRB-approved written informed consent document (Indiana University Purdue University of Indianapolis IRB, #130210786). Collections were performed between 9:00 and 11:00 a.m. The donors were advised to refrain from intake of any food or beverages one hour before the collection. Salivary secretion was stimulated by chewing a gum base for 1-min. Saliva from the 1st min of stimulation was discarded and the following saliva sample was collected directly into ice-chilled tubes, for 2 h. Saliva samples were pooled and immediately centrifuged at 14,000 x *g*, for 20-min, at 4°C. The supernatant was separated from the pellet and stored at -80°C.

### Proteome experiment

#### Specimen preparation and treatment

Thirty-two enamel slabs (8×8×2 mm^3^) were cut, and flattened using sequential water-cooled abrasive discs (500-, 1200-, 2400- and 4000-grit Al_2_O_3_ papers; MD-Fuga, Struers Inc, Cleveland, OH, USA), polished (1-μm diamond suspension, Struers Inc) and sonicated for 3-min in detergent solution. Specimens with any cracks or structure defects were discarded. They were incubated in 2 ml/specimen of whole saliva supernatant for 24 h at room temperature under gentle agitation (85–90 RPM) for AEP formation. Then, all specimens were subjected to 2-min treatments with 4 ml/specimen of the tested rinses, dried with absorbent paper, and immersed in whole saliva supernatant (2 ml/specimen) for 2 h, at room temperature. This cycle was repeated three times.

#### Harvesting AEP from bovine enamel surface specimens

Collection strips of 0.5×1 cm^2^ (Electrode wick filter paper, Bio-Rad, Hercules, CA, USA) pre-soaked in 15.6 mM citric acid were used for AEP collection. The strip was folded and each surface was used to collect AEP from one sample in a standardized manner (4 strip/group). After collection, the strips from each group were placed into a labeled polypropylene microcentrifuge tube and kept frozen at -80°C until analysis [15].

#### AEP protein extraction and in-solution digestion

In-solution digestion from AEP collected material was performed as described previously [15]. Briefly, 200 μL of 50 mM ammonium bicarbonate (pH 7.8) was added to each tube containing the collection strips and then sonicated for 1-min. This procedure was repeated three times. Samples were centrifuged at 14,000 x *g* for 10 minutes and the supernatant was separated and dried by rotary evaporator. The dried material was resuspended in 100 μL of distilled water and a total protein concentration was measured by Micro bicinchoninic acid (Micro BCA) assay. This procedure was repeated for each group. The equivalent of 20 μg of total protein was dried and resuspended using 50 μL of 4 M urea, 10 mM DTT and 50 mM ammonium bicarbonate at pH 7.8 and incubated for 1-h at room temperature. After that, 0.4 μg of 2% (w/w) trypsin (Promega, Madison,WI, USA) in 150 μL of 50 mM ammonium bicarbonate was added to the samples and incubated for at least 16-h at 37°C. The digested protein solutions were dried, de-salted, and subjected to mass spectrometry.

#### Liquid Chromatography Electrospray Ionization Tandem Mass Spectrometry (LC-ESI-MS/MS)

Mass spectrometric analyses were conducted using a LTQ-Velos (Thermo Scientific, San Jose, CA, USA) which allows for in-line liquid chromatography with the capillary fused silica column linked to the mass spectrometer using electrospray ionization in a survey scan in the range of m/z values 390–2000 tandem MS/MS. A dynamic exclusion criterion was established as a repeat count of 1 and a repeat duration of 30 s. All samples were subjected to reversed-phase LC-ESI-MS/MS. A total of 20 μg per sample was subjected to nano-flow reversed-phase nano-HPLC. The nano-flow reversed-phase nano-HPLC was developed with linear 65-min gradient ranging from 5% to 55% of solvent B (97.5% acetonitrile, 0.1% formic acid) at a flow rate of 300 nL/min with a maximum pressure of 280 bar. Electrospray voltage and the temperature of the ion transfer capillary were 2.0 kV and 249°C, respectively. Each survey scan (MS) was followed by automated sequential selection of eight peptides for CID, with dynamic exclusion of the previously selected ions [15].

#### Peptide and protein identification

For peptides, the obtained MS/MS spectra were searched against human protein databases (Swiss Prot and TrEMBL, Swiss Institute of Bioinformatics, Geneva, Switzerland, http://ca.expasy.org/sprot/) using SEQUEST and Percolator algorithms in Proteome Discoverer 1.3 software (Thermo Scientific, San Jose, CA, USA). A maximum of two miscleavages were allowed; Carbamydomethylation of cysteine; phosphorylation of serine, threonine and tyrosine; and oxidation of methionine were included as dynamic modification. While for proteome analysis, trypsin specific cleavage site was considered. Search results were filtered for a False Discovery Rate of 1% employing a decoy search strategy utilizing a reverse database. A total of three mass spectrometric runs were carried out in each condition [15].

#### Integration and relative proteome quantitation

For quantitative proteome analysis, three MS raw files from each group were analyzed using SIEVE technology (Version 2.0 Thermo Scientific, San Jose, CA, USA). Signal processing was performed in a total of 16 MS raw files. The SIEVE experimental workflow was defined as “Control Compare Trend Analysis” where one group of samples was compared to one or more other group of samples. Here the control samples (water treated group) were compared to each of the samples that were harvested after ions treatment. For the alignment step, a single MS raw file belonging to the control group was selected as the reference file and all of the other files were adjusted to generate the best correlation to this reference file. After alignment, the feature detection and integration (or framing) process was performed using the MS level data only. Next, peptide sequences obtained from the database search using SEQUEST algorithm in Proteome Discoverer 1.3 were imported into SIEVE. A filter was applied to the peptide sequences during the import that eliminated all sequences with a Percolator q-value greater than 1% (false discovery rate). Peptides belonging to the same protein were grouped into proteins and a protein ratio and p-value were calculated. Only proteins observed in all four groups were quantified. Control AEP group was used as our default group and all other three groups were compared with the control AEP group. Relative abundance of an individual protein from each group was considered significantly different protein level when the values observed were < 0.75 for decrease abundance or > 1.25 for increase abundance, and a *p*-value < 0.05 as described [11].

#### Enzyme-linked immunosorbent assay (ELISA)

A validation experiment was carried out with one of the proteins identified and relative quantified in the mass spectrometry. ELISA microtiter plate (96-wells) was coated with 100 μl of AEP protein material from each group (10 μg/ml) at 37°C for 1 hour. The plate was then washed three times with 250 μl Tris Buffered Saline (TBS) per well and 200 μl TBST containing 3% BSA added to each well to block uncoated sites, and incubated overnight at 4°C. Primary anti histatin 1 antibody (50 μl; 1:2500 dilution, Abcam, ab97950, MA, USA) in TBST containing 1% BSA was added to each well and incubated at 37°C for 1.5 h, followed by washing three times, and incubation with horse radish peroxidase (HRP) linked anti-rabbit IgG antibody (100μl; 1:30000 dilution, ROCKLAND, PA, USA) in TBST containing 1% BSA. After incubation in the dark for 1 h at room temperature OPD (o-phenylenediamine dihydrochloride, Sigma-Aldrich, MO, USA) was added and product wasanalyzed spectrophotometrically at 490 nm. Histatin 1 levels in each sample were determined by reference to a histatin 1 standard curve and assessed by linear regression analysis. Histatin 1 protein (Chinapeptide, Shanghai, China) purity and M^r^ were determined by mass spectrometry analysis. Analysis of variance and Student-Newman-Keuls test for pairwise comparisons was carried out to compare the values among the groups.

### Functional Experiment

#### Specimen preparation

Forty enamel slabs (4×4×2 mm^3^) were embedded in acrylic resin blocks (Varidur, Buehler, Lake Bluff, IL, USA), then flattened and polished, as described above. Adhesive UPVC tapes were placed on the polished surface of each slab, leaving a central area of 4×1 mm^2^ exposed. Specimens were then randomly assigned to the test groups.

#### Demineralization-cycling procedures

Specimens were treated independently throughout the experiment. In attempt to allow a AEP to form before starting the cycling procedure, specimens were incubated in whole saliva supernatant (2 ml/specimen) for 24-h under gentle agitation, for AEP formation. Then specimens were subjected to cycling procedure, each cycle consisted of 5-min demineralization by immersion in 15.6 mM citric acid (pH 2.6, 4 ml/specimen), followed by 60-min remineralization in whole saliva supernatant (2 ml/specimen), repeated 6×/d, for 5-d. The saliva was renewed 3×/d, while the acid was renewed after each erosion episode. Rinse treatments (4 ml/specimen) were performed for 2-min, 30-min after starting the 1st, 3rd and 6th remineralization periods. Specimens were rinsed with DIW for 10-s after acid exposure and the excess of water gently dried with absorbent paper (Kimwipes, Neenah, WI, USA). All the experimental procedures were conducted at room temperature.

#### Surface profilometry

After cycling, the tapes were removed from the specimens and the surface analyzed. An area 2 mm long (X) ×1 mm wide (Y) was scanned with an optical profilometer (Proscan 2000, Scantron, Venture Way, Tauton, UK). The scan covered the treated area and protected reference surfaces on both sides. The step size was set at 0.01 mm and the number of steps at 200 in the X-axis; and at 0.05 mm and 20, respectively, in the Y-axis. Dedicated software calculated the surface loss of the treated area, by subtracting the average height of the test area from the average height of the two reference surfaces (Proscan Application software v. 2.0.17, Scantron).

### Statistical analysis

ANOVA was used to test the effects of mouthrinse (F, Sn, Sn+F) on surface loss. Pair-wise comparisons were made using Tukey's method to control the overall significance level at 5%.

## Results

### Proteome experiment

**Fig 1:**
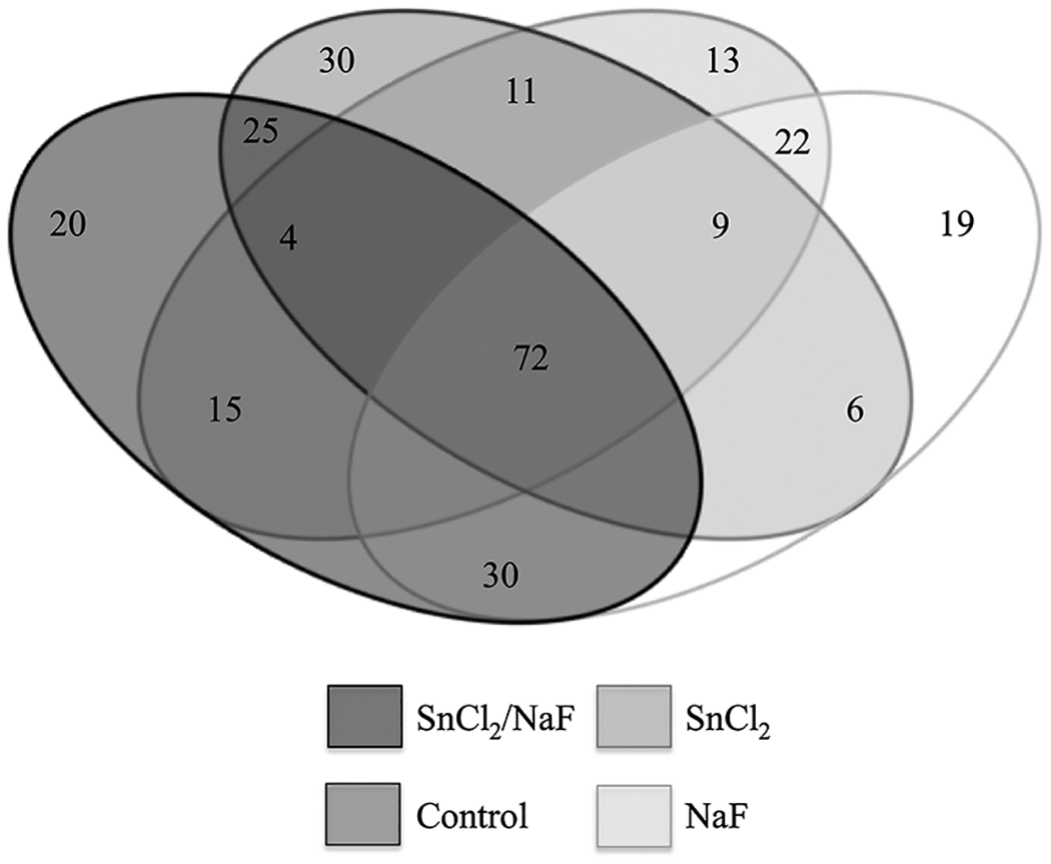
Venn diagram displays numbers of proteins form each group and their overlaps.

A total of 157 proteins were identified in the control group, with 30 proteins exclusively present in this group; in the Sn+F group 166 proteins were identified with 20 proteins exclusively identified in this group. The F group showed a total of 158 proteins with 19 proteins only present in this group. The Sn group showed a total of 146 proteins and 13 only identified for this treatment. A total of 72 proteins were identified in all four groups (Fig 1).

In comparison with control, combination group showed higher abundance of most of common proteins identified in all groups compared to control (Table A in S1 File). Those proteins included Mucins (MUC7 and MUC5B), Albumin, Histatins 1 and 3, acidic PRP 1 and 2, small PRP, and basic PRP, Cystatins C, S, SN, SA, D and A, Protein S100-A8 and 9 and others. For F group, MUC7, histatin 3, and acidic PRP were higher compared to control. Histatin 3 and cystatin D abundance were higher in Sn group compared to control. Nevertheless, statherin showed significant less abundance in groups with isolated ions, while combination group showed no difference compared to control. Annexin A1 was detected in Sn and F groups but not in their combination (Table B in S1 File). Some proteins related to innate immune system were detected exclusively in Sn group as Lactoperoxidase and Serotransferrin. Lysozyme C, Filaggrin and other proteins were detected exclusively in F group.

The results for relative abundance of the common proteins are presented in Table A in S1 File and the protein names identified for each treatment and combinations are described in Tables B-I in S1 File.

**Fig 2:**
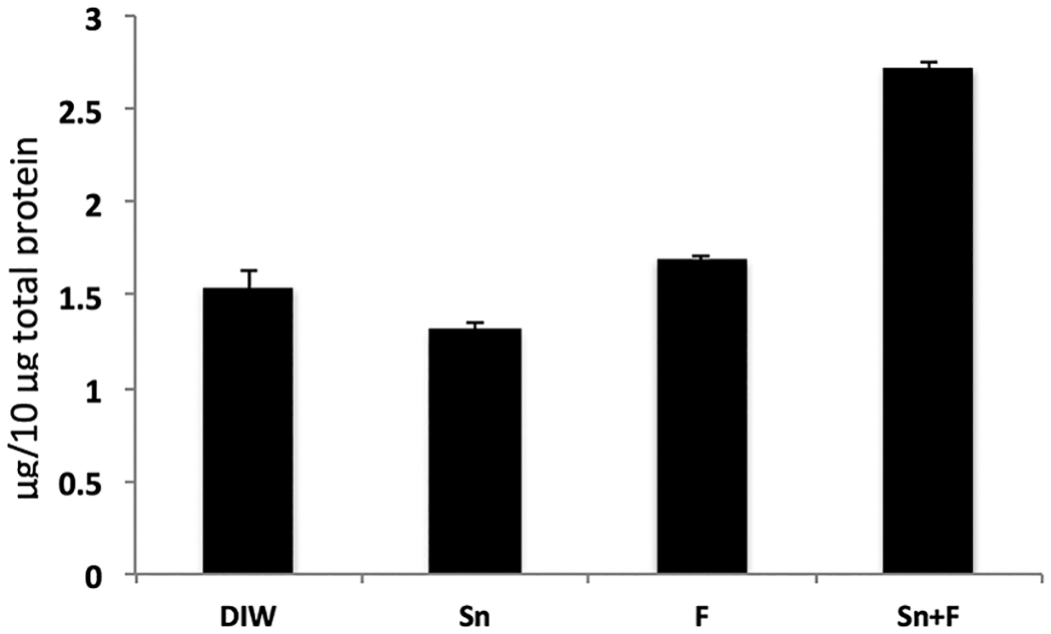
ELISA test of AEP for each group and anti-histatin1 antibody. Means (SD) of histatin 1 in the tested groups.

To validate the differential protein level identified by quantitative mass spectrometry approach, ELISA was carried out on one of the proteins identified, histatin 1. Mass spectrometric analysis observed not significant protein level difference among Sn, F and control group. A significant increased protein level was observed in Sn+F group when compared with the control group. By ELISA histatin 1contents demonstrated to increase from 1.54 ± 0.21 μg/10 μg AEP total protein in the control group, to 2.73 ± 0.15 in SN+F group. While the Sn and F groups were 1.31 ± 0.25 and 1.69 ± 0.41, respectively (Fig 2). The results observed in the ELISA experiment confirm the phenomenon observed when the histatin 1 was analyzed by mass spectrometry.

### Functional experiment

Bovine enamel treated with Sn+F, Sn and F demonstrated a reduction on surface loss by 89%, 67%, and 42% compared with control group (treated with distilled water) respectively. All treatments demonstrated a statistical significance difference (p<0.05). Means and standard deviations are presented in Table 1.

**Table 1:** Mean surface loss (standard-deviation), in μm, for the different rinse treatments used in the study.

Rinses	Sn+F	Sn	F	DIW
**Surface loss**	2.05 (0.72) a	6.14 (1.50) b	10.85 (1.51) c	18.60 (3.32) d

Different letters represent statistically significant differences (p<0.05).

## Discussion

The demineralization-cycling model used in this study was designed to accommodate the formation of AEP on bovine tooth surface and to mimic the pathologic episodes of dental erosion in the dynamic demineralization-remineralization process dictated by AEP (saliva)/tooth interface. Citric acid was used as it is present in most of the acidic beverages available [4–6, 13]. Frequency and length of acid (6×/d, 5 min) and test rinses (3×/d, 2-min) exposures were similar to previously published protocols [13, 16]. For the proteome characterization on bovine enamel surface, a modified protocol was carried out in order to evaluate the influence of the tested ions on AEP proteome formation. The demineralization-cycling model was not included during the proteome exploration, due to the limited amount of AEP proteins that could be harvested after acid exposure. In this study, the treatment sequence was designed according to Veeregowda et al (2011) [17] in which that stannous ion was suggested to have cross-linking action on proteins of previously formed AEP.

The results of this study showed that AEP composition differed significantly depending on the rinse treatments. It has been suggested that salivary proteins play a significant role in the development of dental erosion [18–22]. Therefore, AEP protein composition could be of relevance in modifying the erosion protective mechanism of Sn and F. Higher relative abundance of proteins was found on the Sn+F group, which also presented the higher anti-erosion effect measured our study. Rykke et al. (1991) also observed that the amino acid abundance of *in vivo* enamel AEP was higher in the SnF_2_ treated group compared to the controls [23]. The rationale behind this protective action would be that AEP proteins can influence the formation and stabilization of CaF_2_ precipitates formed on the enamel surface after fluoride exposure, due to the adsorption of phosphates and proteins increasing the efficacy of the fluoride treatment [6, 14, 16, 24]. Some of the proteins observed in higher relative abundance for Sn+F group have been related to the dynamic processes of dental mineral gain and loss.

From the 72 proteins identified in all groups, a group of well-known salivary proteins were notably important due to the recognized function of these proteins on dental erosion episodes [18–20]. Mucins (MUC7 and MUC5B) showed significantly higher abundance in Sn+F group compared to control. These proteins are considered major structural constituents of AEP, providing a physical barrier preventing acids from contacting the tooth surfaces. MUC5B, a protein found in the basal layer of the pellicle, has shown to persist even after 120-min of citric acid exposure [25]. Additionally, MUC5B and MUC7 are characterized with heterotypic complexing property, which may promote interactions with other proteins, such as amylase, further enhancing the protective properties of AEP against erosion and even abrasion [26].

Albumin also showed relatively higher abundance only in Sn+F. It is a hydrophilic acidic protein with a molecular weight in the order of 66 kDa, which has been previously investigated by its potential to inhibit erosion when in acidic solutions [19, 27], although this particular benefit has been questioned [28]. When in the presence of Sn and F, albumin was shown to enhance the protection against erosive and abrasive challenges [29]. The protective effect is thought to be provided by its ability to adsorb to hydroxyapatite [30], contributing to the formation of the AEP, and also to the penetration into the enamel porosities [31]. Some other proteins with strong binding ability to hydroxyapatite, such as acidic PRPs [32], histatin 1 [7], cystatin S [33, 34] and S100-A8 and 9 [35, 36]were also relatively more abundant in the Sn+F group. Perhaps, the presence on Sn may have helped promoting cross-linking among the proteins [17], which could create a more resistant surface protective AEP. It is intriguing, however, that the same proteins were not observed in high abundance in the Sn-only group. Association of Sn to F seems to play a key role explaining the interaction between proteins.

Carbonic anhydrase 6 (CA-VI) was also found in higher relative abundance in the Sn+F group, and it may have some impact in erosive tooth wear prevention, as it has been related to acid neutralization, when present in the pellicle [37]. However, Zwier et.al. (2013) did not show significant difference in the amount of CA-VI in the saliva of erosion and non-erosion subjects [20]. Statherin showed lower abundance in the Sn and F groups, while the abundance of Sn+F was no different from the control. This is somewhat surprising, as statherin has been found to decrease the rate of demineralization [38]. Some other proteins were exclusively detected in the AEP of a single group or in overlap between 2 groups (Tables B-I in S1 File). Annexin A1, despite being a cytoplasmic protein and likely be present in very small amount in saliva was found only in the Sn and F groups and could be of potential interest on the exploration of a group of proteins with biological effect against demineralization process [39]. Annexin 1 is a member of multigene proteins family. It binds to Ca^2+^ and phospholipids and participate in the regulation of Ca^2+^ cytoplasmic concentrations and Ca^2+^ currents across membranes. Therefore, Annexin 1 could be of potential interest in demineralization prevention [40]. In addition, since AEP serves as a solid support for the formation of oral biofilm, a different AEP composition can modulate the formation of those early oral biofilm, effect that likely can influence the development of dental caries.

The functional experiment revealed that the combination of Sn and F provided the best overall protection against ETW being consistent with previous studies [5] and reinforcing some additive effects between these ions. Schlueter et al. (2009) have suggested that this protection results from the formation of more stable and acid-resistant precipitates on the tooth surface, when compared to those formed in the presence of F or Sn alone [16]. The superior protective effect of Sn on enamel compared to F could be explained by the higher inorganic material in enamel since the Sn protection seems to be mainly related to its high reactivity to hydroxyapatite [41].

## Conclusions

Under the limitations of this study, we conclude that AEP proteome varied according to the treatment received. Moreover, all tested rinses were able to reduce enamel erosion progression. An amplified protective effect was observed when Sn and F were combined. This outcome can suggest that the formation of the AEP can contribute to the biological effect related to protection of enamel against erosion.

## Supporting Information

S1 File The relative abundance of proteins present in all groups compared to DIW control (n = 10) (Table A). Proteins present in SnCl_2_ and NaF groups (Table B). Proteins present in SnCl_2_ and SnCl_2_ /NaF groups (Table C). Proteins present in SnCl_2_ /NaF and DIW (control) groups (Table D). Proteins present in NaF and SnCl_2_ /NaF groups (Table E). Proteins exclusively present in SnCl_2_ group (Table F). Proteins exclusively present in NaF group (Table G). Proteins exclusively present in SnCl_2_ / NaF group (Table H). Proteins exclusively present in DIW (control) group (Table I).
(DOC)
