# Supplementary material for: The Impact of Stannous, Fluoride Ions and Its Combination on Enamel Pellicle Proteome and Dental Erosion Prevention
Source: PLoS One. 2015 Jun 1;10(6):e0128196. doi: 10.1371/journal.pone.0128196 (PMC4452394; doi:10.1371/journal.pone.0128196)
Supplement: S1 File — Proteins present in SnCl2 and NaF groups (Table B). Proteins present in SnCl2 and SnCl2 /NaF groups (Table C). Proteins present in SnCl2 /NaF and DIW (control) groups (Table D). Proteins present in NaF and SnCl2 /NaF groups (Table E). Proteins exclusively present in SnCl2 group (Table F). Proteins exclusively present in NaF group (Table G). Proteins exclusively present in SnCl2 / NaF group (Table H). Proteins exclusively present in DIW (control) group (Table I). (DOC) [file pone.0128196.s001.doc]

Table S1A. the relative abundance of proteins present in all groups compared to DIW control (n=8). (Sn: SnCl_2_, F: NaF, Sn+F: SnCl_2_/NaF, DIW: deionized water)

| **Accession Number** | **Protein Name** | **Sn/DIW** | **P Value** | **F/DIW** | **P Value** | **Sn+F/DIW** | **P Value** |
| --- | --- | --- | --- | --- | --- | --- | --- |
| **A7Y9J9** | Mucin 5AC, oligomeric mucus/gel-forming ^l^ | 1.20 | 0.001 | 2.38 | 0.278 | 1.98 | 0.000 |
| **A8K2H9** | cDNA FLJ78503, highly similar to Homo sapiens keratin 13 (KRT13), transcript variant 1, mRNA ^l^ | 0.90 | 0.001 | 0.92 | 0.001 | 1.87 | 0.000 |
| **A8K2U0** | Alpha-2-macroglobulin-like protein 1 ^a^ | 1.05 | 0.001 | 0.96 | 0.002 | 1.32 | 0.000 |
| **B1AN48** | Small proline-rich protein 3 ^a,b,j^ | 0.56 | 0.001 | 0.99 | 0.000 | 1.87 | 0.010 |
| **B4DGT4** | cDNA FLJ61241, highly similar to Histone deacetylase 5 ^a,b^ | 0.87 | 0.001 | 0.94 | 0.020 | 1.38 | 0.013 |
| **B4DPR2** | cDNA FLJ50830, highly similar to Serum albumin ^d^ | 0.90 | 0.001 | 1.14 | 0.002 | 1.77 | 0.045 |
| **B4DZ16** | cDNA FLJ58649 ^l^ | 0.53 | 0.001 | 1.02 | 0.004 | 1.42 | 0.320 |
| **B4E1T1** | cDNA FLJ54081, highly similar to Keratin, type II cytoskeletal 5 ^l^ | 1.13 | 0.001 | 1.07 | 0.031 | 1.37 | 0.015 |
| **B5ME49** | Mucin-16 ^l^ | 0.97 | 0.001 | 0.93 | 0.001 | 1.09 | 0.020 |
| **B7Z5K0** | cDNA FLJ52445, highly similar to Homo sapiens membrane-associated ring finger (C3HC4) 7 (MARCH7), mRNA ^m^ | 1.97 | 0.001 | 1.98 | 0.070 | 2.50 | 0.001 |
| **B7ZMD7** | Amylase, alpha 1A (Salivary) ^a,m^ | 1.19 | 0.001 | 0.65 | 0.000 | 1.20 | 0.003 |
| **B8ZZJ3** | Alstrom syndrome protein 1 ^l^ | 0.99 | 0.001 | 1.01 | 0.017 | 1.34 | 0.002 |
| **C9JA77** | Uncharacterized protein ^l^ | 0.69 | 0.001 | 0.65 | 0.000 | 2.32 | 0.001 |
| **E7EMQ1** | Carbonic anhydrase 6 ^a,d,m^ | 0.90 | 0.001 | 1.01 | 0.038 | 1.38 | 0.002 |
| **E7EQT2** | Mucin-4 beta chain ^g^ | 0.91 | 0.001 | 0.91 | 0.003 | 1.37 | 0.003 |
| **E7ESM2** | Urokinase-type plasminogen activator chain B ^l^ | 0.88 | 0.001 | 0.86 | 0.003 | 1.34 | 0.000 |
| **E7ETI5** | Uncharacterized protein ^l^ | 0.99 | 0.001 | 1.18 | 0.001 | 1.43 | 0.002 |
| **F4MHJ1** | Ubiquitously transcribed tetratricopeptide repeat protein Y-linked transcript variant 97  ^b,m^ | 0.95 | 0.001 | 0.98 | 0.001 | 1.43 | 0.005 |
| **F6KPG5** | Albumin ^a,b,d,h,j,m^ | 0.92 | 0.001 | 0.84 | 0.001 | 1.45 | 0.012 |
| **G3CIG0** | MUC19 variant 12 ^l^ | 0.97 | 0.001 | 0.98 | 0.001 | 1.46 | 0.001 |
| **H0Y930** | Extracellular matrix protein FRAS1 ^e^ | 0.96 | 0.001 | 0.69 | 0.001 | 1.08 | 0.001 |
| **H6VRF8** | Keratin 1 ^a,b,c^ | 0.81 | 0.001 | 0.93 | 0.001 | 1.67 | 0.006 |
| **H7BXM7** | Uncharacterized protein ^l^ | 1.10 | 0.001 | 1.52 | 0.001 | 1.61 | 0.003 |
| **H7BYJ0** | Uncharacterized protein ^l^ | 0.92 | 0.001 | 0.87 | 0.001 | 1.60 | 0.005 |
| **O15079** | Syntaphilin ^j^ | 0.97 | 0.001 | 1.17 | 0.001 | 1.48 | 0.001 |
| **P01034** | Cystatin-C ^a,c^ | 1.08 | 0.002 | 0.92 | 0.001 | 1.37 | 0.000 |
| **P01036** | Cystatin-S ^a,b,n^ | 0.70 | 0.002 | 0.99 | 0.001 | 1.51 | 0.000 |
| **P01037** | Cystatin-SN ^a^ | 0.98 | 0.001 | 0.95 | 0.001 | 1.54 | 0.002 |
| **P01833** | Polymeric immunoglobulin receptor ^c,k^ | 1.06 | 0.000 | 1.20 | 0.001 | 1.40 | 0.002 |
| **P01877** | Ig alpha-2 chain C region ^c^ | 0.99 | 0.010 | 1.06 | 0.001 | 1.38 | 0.002 |
| **P02808** | Statherin ^b,h,i,m^ | 0.59 | 0.030 | 0.57 | 0.001 | 1.08 | 0.000 |
| **P02810** | Salivary acidic proline-rich phosphoprotein ½ ^b,h,m^ | 0.93 | 0.017 | 2.25 | 0.001 | 2.37 | 0.023 |
| **P02812** | Basic salivary proline-rich protein 2 ^a,b,c,i^ | 1.16 | 0.002 | 0.99 | 0.001 | 1.43 | 0.000 |
| **P02814** | Submaxillary gland androgen-regulated protein 3B ^l^ | 1.11 | 0.048 | 1.00 | 0.001 | 1.40 | 0.001 |
| **P04080** | Cystatin-B ^a,b,n^ | 0.90 | 0.048 | 0.55 | 0.001 | 1.19 | 0.006 |
| **P06733** | Alpha-enolase ^a,b^ | 0.55 | 0.002 | 0.71 | 0.002 | 1.42 | 0.002 |
| **P07737** | Profilin-1 ^g,j^ | 1.05 | 0.003 | 1.25 | 0.001 | 1.42 | 0.001 |
| **P09228** | Cystatin-SA ^a,k^ | 0.98 | 0.019 | 0.97 | 0.001 | 1.35 | 0.004 |
| **P0CG05** | Ig lambda-2 chain C regions ^b,c^ | 0.75 | 0.051 | 0.62 | 0.001 | 1.10 | 0.006 |
| **P15515** | Histatin-1 ^b,h,n^ | 0.89 | 0.068 | 0.78 | 0.001 | 1.46 | 0.002 |
| **P15516** | Histatin-3 ^c,h,n^ | 3.20 | 0.005 | 1.96 | 0.001 | 3.27 | 0.001 |
| **P19961** | Alpha-amylase 2B ^a^ | 0.65 | 0.010 | 0.96 | 0.001 | 1.23 | 0.003 |
| **P28325** | Cystatin-D ^a^ | 1.54 | 0.002 | 0.91 | 0.001 | 1.28 | 0.001 |
| **P35908** | Keratin, type II cytoskeletal 2 epidermal ^b,j^ | 0.79 | 0.350 | 0.78 | 0.001 | 1.41 | 0.001 |
| **P54652** | Heat shock-related 70 kDa protein 2 ^k^ | 0.93 | 0.015 | 0.78 | 0.001 | 2.35 | 0.001 |
| **Q8TAX7** | Mucin-7 ^a^ | 0.93 | 0.000 | 2.02 | 0.001 | 1.47 | 0.002 |
| **Q8WVD6** | PHTF2 protein ^l^ | 0.75 | 0.825 | 0.89 | 0.001 | 1.48 | 0.005 |
| **Q8WZ42** | Titin ^a,b,k^ | 0.93 | 0.095 | 0.92 | 0.001 | 1.37 | 0.001 |
| **Q9HC84** | Mucin-5B ^a,i^ | 0.79 | 0.278 | 0.91 | 0.001 | 2.36 | 0.001 |
| **Q9NR09** | Baculoviral IAP repeat-containing protein 6 ^a,b,e,f^ | 0.95 | 0.001 | 0.79 | 0.001 | 1.47 | 0.002 |
| **Q9NV58** | E3 ubiquitin-protein ligase RNF19A ^b,k,m^ | 0.62 | 0.000 | 1.95 | 0.001 | 1.14 | 0.001 |
| **A0M8Q9** | C1 segment protein ^a,b,c^ | 0.96 | 0.234 | 0.78 | 0.001 | 1.58 | 0.001 |
| **A8K5I6** | cDNA FLJ78643, highly similar to Homo sapiens cornulin (CRNN), mRNA ^h,j,m^ | 0.91 | 0.020 | 0.94 | 0.001 | 1.44 | 0.012 |
| **B2R853** | cDNA, FLJ93744, highly similar to Homo sapiens keratin 6E (KRT6E), mRNA ^l^ | 1.11 | 0.002 | 0.97 | 0.001 | 1.39 | 0.012 |
| **B3W5Y6** | Serpin B3 ^a^ | 1.06 | 0.002 | 0.95 | 0.001 | 1.48 | 0.004 |
| **B4DL17** | cDNA FLJ52558, highly similar to Keratin, type I cytoskeletal 13 ^a^ | 1.85 | 0.003 | 1.37 | 0.015 | 1.33 | 0.001 |
| **B4DRR0** | cDNA FLJ53910, highly similar to Keratin, type II cytoskeletal 6A ^b,j^ | 0.89 | 0.002 | 1.00 | 0.003 | 1.96 | 0.002 |
| **E7EQV7** | Uncharacterized protein ^l^ | 0.95 | 0.002 | 0.92 | 0.032 | 1.49 | 0.001 |
| **G3V1A4** | Cofilin 1 (Non-muscle), isoform CRA ^b,e^ | 0.62 | 0.001 | 1.01 | 0.006 | 1.30 | 0.011 |
| **P01040** | Cystatin-A ^a,h,g,n^ | 0.98 | 0.017 | 0.86 | 0.171 | 1.26 | 0.023 |
| **P01857** | Ig gamma-1 chain C region ^c^ | 0.94 | 0.000 | 0.90 | 0.028 | 1.34 | 0.010 |
| **P04792** | Heat shock protein beta-1 ^k^ | 1.09 | 0.038 | 1.91 | 0.002 | 2.61 | 0.010 |
| **P05109** | Protein S100-A8 ^c,e,h,m,n^ | 0.74 | 0.041 | 0.95 | 0.002 | 1.35 | 0.015 |
| **P06702** | Protein S100-A9 ^c,e,h,m,n^ | 0.83 | 0.001 | 0.88 | 0.003 | 1.39 | 0.003 |
| **P07355** | Annexin A2 ^j, k,m^ | 1.15 | 0.024 | 1.23 | 0.001 | 1.47 | 0.016 |
| **P27482** | Calmodulin-like protein 3 ^h,m^ | 0.80 | 0.003 | 1.01 | 0.000 | 1.42 | 0.001 |
| **P31947** | 14-3-3 protein sigma ^a,b,f,k^ | 0.94 | 0.002 | 0.95 | 0.035 | 1.45 | 0.002 |
| **P47929** | Galectin-7 ^f,g^ | 0.84 | 0.003 | 0.78 | 0.001 | 1.39 | 0.003 |
| **P62805** | Histone H4 ^e^ | 1.06 | 0.003 | 2.96 | 0.005 | 2.32 | 0.000 |
| **P68371** | Tubulin beta-4B chain ^a,b,e^ | 1.25 | 0.005 | 0.99 | 0.005 | 1.42 | 0.000 |

^a^ Homeostasis/Metabolism, ^b^ Protein binding/synthesis/modification , ^c^ Immune response , ^d^ Transportation/ metabolites transporter , ^e^ Cell cycle/signaling/ growth, ^f^ Apoptosis, ^g^ Cytoskeletal/ECM/adhesion, ^h^ Biomineralization, ^i^ Lubrication, ^j^ Tissue regeneration, ^k^ Other biological functions, ^l^ Unknown biological function, ^m^ Calcium/phosphates/metal-binding, ^n^ antimicrobial.

Table S1B. Proteins present in SnCl_2_ and NaF groups.

| **Accession Number** | **Protein Name** |
| --- | --- |
| A4D1R9 | Homeodomain interacting protein kinase 2  b,e,j,k |
| B3KVI8 | cDNA FLJ16604 fis, clone TESTI4008097, highly similar to Polycomb group protein ASXL1^b^ |
| B3KVV3 | cDNA FLJ41584 fis, clone CTONG2020445, highly similar to ATP-binding cassette sub-family A member 12 ^b^ |
| B4DT53 | cDNA FLJ52905, highly similar to Runt-related transcription factor 3 ^b^ |
| B4E1M1 | cDNA FLJ60391, highly similar to Lactoperoxidase (EC 1.11.1.7) ^c^ |
| D3DVP6 | Macrophage erythroblast attacher, isoform CRA_d  ^l^ |
| E7EQE3 | Uncharacterized protein ^l^ |
| E7ESP5 | Uncharacterized protein ^l^ |
| E7EWI7 | Uncharacterized protein ^l^ |
| F8WAI1 | Immunoglobulin-like and fibronectin type III domain-containing protein 1 ^l^ |
| H7C0P6 | Mitogen-activated protein kinase kinase kinase kinase 4 ^b^ |
| O43157 | Plexin-B1 ^e,g,j^ |
| P49454 | Centromere protein F ^b,e,g,j^ |
| Q2NKL1 | Mineralocorticoid receptor ^b,d,k,m^ |
| Q5T1R4 | Transcription factor HIVEP3 ^b,e,j,m^ |
| Q5T3N0 | Annexin A1 ^a,b,c,e,j,m^ |
| Q6WKZ4 | Rab11 family-interacting protein 1 ^b,e,c^ |
| Q6WRI0 | Immunoglobulin superfamily member 10 ^b,e,j^ |
| Q7Z7G8 | Vacuolar protein sorting-associated protein 13B ^b^ |
| Q9BQK8 | Phosphatidate phosphatase LPIN3 ^a,k,m^ |
| Q9UKZ4 | Teneurin-1 b,e,c,g,j |
| Q9ULK2 | Ataxin-7-like protein 1 ^l^ |

^a^ Homeostasis/Metabolism, ^b^ Protein binding/synthesis/modification , ^c^ Immune response , ^d^ Transportation/ metabolites transporter , ^e^ Cell-cycle/signaling/ growth, ^f^ Apoptosis, ^g^ Cytoskeletal/ECM/adhesion, ^h^ Biomineralization, ^i^ Lubrication, ^j^ Tissue regeneration, ^k^ Other biological functions, ^l^ Unknown biological function, ^m^ Calcium/phosphates/metal-binding, ^n^ antimicrobial.

Table S1C. Proteins present in SnCl_2_ and SnCl_2_ /NaF groups.

| **Accession Number** | **Protein Name** |
| --- | --- |
| Q9C0A1 | Zinc finger homeobox protein 2 ^b,m^ |
| A8K008 | cDNA FLJ78387 ^l^ |
| A8K4G7 | cDNA FLJ78528, highly similar to Homo sapiens vacuolar protein sorting 4B (yeast) (VPS4B), mRNA ^k^ |
| B3KU50 | cDNA FLJ39199 fis, clone OCBBF2005189, highly similar to Homo sapiens ankyrin repeat domain 17 (ANKRD17), transcript variant 2, mRNA ^k^ |
| B4DII4 | cDNA FLJ53493, highly similar to HEF-like protein |
| B5MCY1 | Tudor domain-containing protein 15 ^b^ |
| C0JYZ1 | Dynein, axonemal, heavy chain 11 ^k^ |
| E9PCX8 | Tensin-3 ^e,m^ |
| F5H2N0 | Uncharacterized protein l |
| O60284 | Suppression of tumorigenicity 18 protein ^b,m^ |
| P35568 | Insulin receptor substrate 1 ^a,b,c,e,j,k^ |
| Q53EU2 | GATA binding protein 6 variant b,m |
| Q9NSI6 | Bromodomain and WD repeat-containing protein 1 ^b,e,g^ |
| Q9ULM3 | YEATS domain-containing protein 2 ^b,e^ |
| Q9UQB3 | Catenin delta-2 ^b,e,g,j^ |

^a^ Homeostasis/Metabolism, ^b^ Protein binding/synthesis/modification , ^c^ Immune response , ^d^ Transportation/ metabolites transporter , ^e^ Cell cycle/signaling/ growth, ^f^ Apoptosis, ^g^ Cytoskeletal/ECM/adhesion, ^h^ Biomineralization, ^i^ Lubrication, ^j^ Tissue regeneration, ^k^ Other biological functions, ^l^ Unknown biological function, ^m^ Calcium/phosphates/metal-binding, ^n^ antimicrobial.

Table S1D. Proteins present in SnCl_2_ /NaF and DIW (control) groups.

| **Accession Number** | **Protein Name** |
| --- | --- |
| A4FUT8 | JMJD1B protein ^l^ |
| A5YKK5 | KIAA0232 l |
| B3KNA1 | cDNA FLJ14021 fis, clone HEMBA1002513, highly similar to Histone deacetylase 6 ^k^ |
| B3KRV8 | cDNA FLJ34970 fis, clone NTONG2005363, highly similar to Castor homolog 1 zinc finger protein ^m^ |
| B4DZR3 | cDNA FLJ59826, highly similar to Zinc finger protein ZFPM m |
| B7Z8W3 | cDNA FLJ53272, highly similar to Homo sapiens LIM domain 7 (LMO7), mRNA ^m^ |
| E9PDX3 | Kinesin-like protein KIF13A ^l^ |
| F6SWM5 | C-Jun-amino-terminal kinase-interacting protein 2 ^l^ |
| F8W7E2 | Uncharacterized protein ^l^ |
| F8WC76 | Uncharacterized protein ^l^ |
| H0Y8W5 | Rho GTPase-activating protein 21 ^e^ |
| H3BLS7 | Vacuolar protein sorting-associated protein 13D ^l^ |
| Q14517 | Protocadherin Fat 1 ^e,g,m^ |
| Q4VXP2 | Potassium voltage-gated channel subfamily KQT member 2 ^l^ |
| Q5CZC0 | Fibrous sheath-interacting protein 2 ^l^ |
| Q6UB99 | Ankyrin repeat domain-containing protein 11 ^a,e,j^ |
| Q6UX82 | Ly6/PLAUR domain-containing protein 8 ^l^ |
| Q6ZMI9 | cDNA FLJ23911 fis, clone CAE01964, highly similar to Homo sapiens EGF, latrophilin and seven transmembrane domain containing 1 (ELTD1) ^e^ |
| Q86YR6 | POTE ankyrin domain family member D ^l^ |
| Q8N7Z5 | Putative ankyrin repeat domain-containing protein 31 ^l^ |
| Q8TB46 | ANKRD50 protein ^l^ |
| Q92574 | Hamartin ^b,d,e,g,j^ |
| Q9UQ35 | Serine/arginine repetitive matrix protein 2 ^b,k^ |
| Q9Y4B6 | Protein VPRBP ^b,e,j,k^ |
| Q9Y6X0 | SET-binding protein ^k^ |

^a^ Homeostasis/Metabolism, ^b^ Protein binding/synthesis/modification , ^c^ Immune response , ^d^ Transportation/ metabolites transporter , ^e^ Cell cycle/signaling/ growth, ^f^ Apoptosis, ^g^ Cytoskeletal/ECM/adhesion, ^h^ Biomineralization, ^i^ Lubrication, ^j^ Tissue regeneration, ^k^ Other biological functions, ^l^ Unknown biological function, ^m^ Calcium/phosphates/metal-binding, ^n^ antimicrobial.

Table S1E. Proteins present in NaF and SnCl_2_ /NaF groups.

| **Accession Number** | **Protein Name** |
| --- | --- |
| A4D1A8 | Similar to Piccolo protein (Aczonin) l |
| A7E2D6 | NAV2 protein ^l^ |
| B4DI39 | cDNA FLJ54328, highly similar to Heat shock 70 kDa protein 1 ^k^ |
| B4DSK7 | cDNA FLJ50196, highly similar to Peroxisome proliferator-activated receptor-binding protein ^b^ |
| B4DV38 | PAP-associated domain-containing protein 5 ^b,e,g,m^ |
| D3DS86 | E3 ubiquitin-protein ligase HECTD1 ^b,j^ |
| D3DX93 | HCG1745555, isoform CRA_b ^l^ |
| E7EPM4 | Mucin-17 ^l^ |
| E9PJL5 | Putative uncharacterized protein C12orf63 ^l^ |
| F5GWX1 | Uncharacterized protein ^l^ |
| F8VW64 | RNA-binding protein Nova-1 ^a,k^ |
| O75592 | Probable E3 ubiquitin-protein ligase MYCBP2 ^b,e,g,j,m^ |
| P15822 | Zinc finger protein 40 ^b,m^ |
| P48169 | Gamma-aminobutyric acid receptor subunit alpha-4 ^d,e,j,k^ |
| P52948 | Nuclear pore complex protein Nup98-Nup96 ^a,b,d,e^ |
| P58397 | A disintegrin and metalloproteinase with thrombospondin motifs 12 ^a,e,g,j,m^ |
| P78333 | Glypican-5 ^a,k^ |
| Q05BP9 | OLIG2 protein ^b,e,j^ |
| Q12923 | Tyrosine-protein phosphatase non-receptor type 13 ^b,e^ |
| Q14204 | Cytoplasmic dynein 1 heavy chain 1 ^b,d,e,k^ |
| Q1RMC5 | Claspin homolog (Xenopus laevis) ^l^ |
| Q2NKW8 | Adenosylhomocysteinase ^a^ |
| Q6N030 | Putative uncharacterized protein DKFZp686I15212 l |
| Q7Z4S6 | Kinesin-like protein KIF21A ^a,k^ |
| Q7Z6E9 | E3 ubiquitin-protein ligase RBBP6 ^b,j,m^ |
| Q86T35 | Putative uncharacterized protein DKFZp451A177 ^m^ |
| Q8WVD6 | PHTF2 protein ^l^ |
| Q9H8V3 | Protein ECT2 ^b,d,e,f,j^ |
| Q9Y2I7 | 1-phosphatidylinositol 3-phosphate 5-kinase ^a,b,e,k,m^ |
| Q9Y6E7 | NAD-dependent protein deacetylase sirtuin-4 ^a,e,m^ |

^a^ Homeostasis/Metabolism, ^b^ Protein binding/synthesis/modification , ^c^ Immune response , ^d^ Transportation/ metabolites transporter , ^e^ Cell cycle/signaling/ growth, ^f^ Apoptosis, ^g^ Cytoskeletal/ECM/adhesion, ^h^ Biomineralization, ^i^ Lubrication, ^j^ Tissue regeneration, ^k^ Other biological functions, ^l^ Unknown biological function, ^m^ Calcium/phosphates/metal-binding, ^n^ antimicrobial.

Table S1F. Proteins exclusively present in SnCl_2_ group.

| **Accession Number** | **Protein Name** |
| --- | --- |
| Q96E61 | Uncharacterized protein ^l^ |
| P06310 | Ig kappa chain V-II region RPMI 6410 ^c,e^ |
| Q6PIT5 | Uncharacterized protein ^l^ |
| Q6GMW0 | Uncharacterized protein ^l^ |
| Q7Z2U7 | Uncharacterized protein ^l^ |
| Q6GMV8 | Uncharacterized protein ^l^ |
| P22079 | Lactoperoxidase ^a,c,k,m^ |
| Q6NS95 | Uncharacterized protein ^l^ |
| Q8NEJ1 | Uncharacterized protein ^l^ |
| Q6PIH6 | Uncharacterized protein ^l^ |
| Q6GMW4 | Uncharacterized protein ^l^ |
| Q6GMX4 | Uncharacterized protein ^l^ |
| P02787 | Serotransferrin ^a,d,k,m^ |

^a^ Homeostasis/Metabolism, ^b^ Protein binding/synthesis/modification , ^c^ Immune response , ^d^ Transportation/ metabolites transporter , ^e^ Cell cycle/signaling/ growth, ^f^ Apoptosis, ^g^ Cytoskeletal/ECM/adhesion, ^h^ Biomineralization, ^i^ Lubrication, ^j^ Tissue regeneration, ^k^ Other biological functions, ^l^ Unknown biological function, ^m^ Calcium/phosphates/metal-binding, ^n^ antimicrobial.

Table S1G. Proteins exclusively present in NaF group.

| **Accession Number** | **Protein Name** |
| --- | --- |
| Q502W4 | Uncharacterized protein ^l^ |
| P61626 | Lysozyme C ^b,c,e,n^ |
| Q6GMV7 | Uncharacterized protein ^l^ |
| Q9UBC9 | Small proline-rich protein 3 ^b,e,j^ |
| Q6PIH4 | Uncharacterized protein ^l^ |
| P14618 | Pyruvate kinase isozymes M1/M2 ^a,b,c,f,m^ |
| P12273 | Prolactin-inducible protein ^a,b,c^ |
| Q8TDL5 | BPI fold-containing family B member 1 ^c,k^ |
| Q71V83 | Alpha-A-crystallin ^k^ |
| C9JS40 | Uncharacterized protein ^l^ |
| B3KSF4 | Probable ATP-dependent RNA helicase DDX4 ^e,j,k^ |
| O15230 | Laminin subunit alpha-5 ^b,e,g,j,k^ |
| B3KU03 | cDNA FLJ39022 fis, clone NT2RP7003724, weakly similar to Serine/arginine repetitive matrix protein 1 ^g^ |
| B4DYM8 | cDNA FLJ60373, highly similar to Zinc finger CCCH domain-containing protein11A ^m^ |
| Q9ULK2 | Ataxin-7-like protein 1 ^l^ |
| F8WEP2 | AEP-ribosylation factor-like protein 6-interacting protein 4 ^l^ |
| E5RJ68 | AP-3 complex subunit beta-1 ^d,e^ |
| F5H894 | Uncharacterized protein ^l^ |
| P20930 | Filaggrin ^e,j,m^ |

^a^ Homeostasis/Metabolism, ^b^ Protein binding/synthesis/modification , ^c^ Immune response , ^d^ Transportation/ metabolites transporter , ^e^ Cell cycle/signaling/ growth, ^f^ Apoptosis, ^g^ Cytoskeletal/ECM/adhesion, ^h^ Biomineralization, ^i^ Lubrication, ^j^ Tissue regeneration, ^k^ Other biological functions, ^l^ Unknown biological function, ^m^ Calcium/phosphates/metal-binding, ^n^ antimicrobial.

Table S1H. Proteins exclusively present in SnCl_2_ / NaF group.

| **Accession Number** | **Protein Name** |
| --- | --- |
| Q6GMX8 | Uncharacterized protein ^l^ |
| P23280 | Carbonic anhydrase 6 ^a,d,k^ |
| P07339 | Cathepsin D ^a,g^ |
| P29508 | Serpin B3 ^a,b,k^ |
| Q9UBX7 | Kallikrein-11 a,b |
| P30740 | Leukocyte elastase inhibitor ^a,b^ |
| Q6PJF2 | Uncharacterized protein ^l^ |
| A6NN68 | Uncharacterized protein ^l^ |
| P02765 | Alpha-2-HS-glycoprotein ^c,e,h,,j^ |
| P13796 | Plastin-2 ^b,c,e,g,m^ |
| P09228 | Cystatin-SA ^a,b^ |
| P02679 | Fibrinogen gamma chain ^b,e,g,j,k,m^ |
| Q13349 | Integrin alpha-D ^c,e,g,m^ |
| H0YET1 | Liprin-beta-2 ^e,g^ |
| B7Z9B9 | Anoctamin ^l^ |
| Q5T6C4 | Ataxin-7-like protein 2 ^l^ |
| D1MPS6 | Uncharacterized protein ^l^ |
| H0Y7L2 | Dedicator of cytokinesis protein 7 ^e,k^ |
| Q5T4S7 | E3 ubiquitin-protein ligase UBR4 ^b,m^ |
| Q9NXG0 | Centlein ^b,k^ |

^a^ Homeostasis/Metabolism, ^b^ Protein binding/synthesis/modification , ^c^ Immune response , ^d^ Transportation/ metabolites transporter , ^e^ Cell cycle/signaling/ growth, ^f^ Apoptosis, ^g^ Cytoskeletal/ECM/adhesion, ^h^ Biomineralization, ^i^ Lubrication, ^j^ Tissue regeneration, ^k^ Other biological functions, ^l^ Unknown biological function, ^m^ Calcium/phosphates/metal-binding, ^n^ antimicrobial.

Table S1I. Proteins exclusively present in DIW (control) group.

| **Accession Number** | **Protein Name** |
| --- | --- |
| Q6P5S2 | UPF0762 protein C6orf58 ^l^ |
| Q5NV90 | V2-17 protein ^c,e^ |
| Q14515 | SPARC-like protein 1 ^e,m^ |
| P31025 | Lipocalin-1 ^b,d,k^ |
| P10599 | Thioredoxin a,b,c,d,e,k |
| Q9NQ38 | Serine protease inhibitor Kazal-type 5 ^b,c,e,j,k^ |
| P31947 | 14-3-3 protein sigma a,b,e,f,j,k |
| P04075 | Fructose-bisphosphate aldolase A ^a,b,e,k^ |
| P00450 | Ceruloplasmin d,m |
| P02749 | Beta-2-glycoprotein 1 ^a,d,e,k^ |
| P20061 | Transcobalamin-1 a,m |
| P60174 | Triosephosphate isomerase ^a,e,k^ |
| P54108 | Cysteine-rich secretory protein 3 ^c^ |
| P09211 | Glutathione S-transferase P ^a,k^ |
| P22748 | Carbonic anhydrase 4 a,d,j,k,m |
| P62937 | Peptidyl-prolyl cis-trans isomerase A ^b,e,k^ |
| P02675 | Fibrinogen beta chain ^b,c,e,g,k^ |
| Q6MZM9 | Uncharacterized protein C4orf40 ^l^ |
| P15516 | Histatin-3 ^h,m,n^ |
| P01009 | Alpha-1-antitrypsin ^a,k^ |
| P06733 | Alpha-enolase ^a,b,e,k,m^ |
| P00738 | Haptoglobin ^a,c,f,k,n^ |
| P02766 | Transthyretin ^a,d,g,k^ |
| B4DYR3 | cDNA FLJ60976 ^l^ |
| Q3MIV8 | Myosin-11 ^j,k^ |
| Q9UPR6 | Zinc finger RNA-binding protein 2 ^k,m^ |
| H7BXJ7 | Uncharacterized protein ^l^ |
| H7BYT2 | Uncharacterized protein ^l^ |
| Q68D65 | Putative uncharacterized protein DKFZp686B17277 ^l^ |
| B4DSN8 | cDNA FLJ60863, highly similar to High mobility group protein 2-like 1 ^l^ |

^a^ Homeostasis/Metabolism, ^b^ Protein binding/synthesis/modification , ^c^ Immune response , ^d^ Transportation/ metabolites transporter , ^e^ Cell cycle/signaling/ growth, ^f^ Apoptosis, ^g^ Cytoskeletal/ECM/adhesion, ^h^ Biomineralization, ^i^ Lubrication, ^j^ Tissue regeneration, ^k^ Other biological functions, ^l^ Unknown biological function, ^m^ Calcium/phosphates/metal-binding, ^n^ antimicrobial.
